# GLP-1 Receptor Agonists – Good for Body Weight, Bad for Micronutrient Status?

**DOI:** 10.1016/j.cdnut.2025.107587

**Published:** 2025-10-30

**Authors:** Felix Kerlikowsky, Klaus Krämer, Manfred Eggersdorfer, Andreas Hahn

**Affiliations:** 1 Institute of Food Science and Human Nutrition, Leibniz University, Hannover, Germany; 2 Society for Applied Vitamin Research e.V. (GVF), Jena, Germany; 3 Johns Hopkins Bloomberg School of Public Health, Baltimore, MD, United States; 4 Department of Internal Medicine, University Medical Center Groningen, Groningen, the Netherlands

**Keywords:** GLP-1 receptor agonists, appetite suppression, weight loss, dietary pattern, nutrient intake, micronutrient status

## Abstract

Obesity is a global health issue with a prevalence predicted to affect 24% of the world's population by 2035. A high-caloric intake and a sedentary lifestyle are, along with other factors, the main causes of obesity. Since 2014, the pharmacological use of glucagon-like peptide-1 receptor agonists (GLP-1 RAs), which mimic the gut hormone GLP-1, has provided an alternative to lifestyle interventions for tackling obesity. GLP-1 RAs suppress appetite and slow gastrointestinal transit time. This reduces food and energy intake to a degree that previous pharmacotherapies have not achieved. Mechanistically, GLP-1 RAs act through peripheral and central nervous pathways, increasing insulin secretion while inhibiting glucagon release and gastric motility. The significant reduction in food consumption raises concerns about adequate dietary intakes with an increased risk of micronutrient deficiencies, particularly among obese individuals. Here, we highlight the need for further research by summarizing current evidence on GLP-1 RA and its potential impact on dietary patterns and micronutrient status. Relevant publications were identified through searches in PubMed/Medline, Scopus, Cochrane Databases, Google Scholar, Web of Science, and Embase databases up to June 2025. Only peer-reviewed articles published in English were considered. We found that the clinical use of GLP-1 RA reduces energy intake by ≤40%, resulting in notable weight loss compared with traditional lifestyle modifications. Consequently, professional dietary counseling is essential in order to support the selection and consumption of nutrient-dense, protein-rich foods and prevent nutrient inadequacies and loss of muscle mass during weight loss. However, given its widespread use in the general population, many individuals using GLP-1 RA may not be under supervision. This could markedly increase risk of micronutrient deficiencies, which are already more prevalent in obesity. Future research is needed to systematically investigate the long-term nutritional consequences of GLP-1 RA, paying particular attention to micronutrient intake and status.

## Introduction

Overweight and obesity are a worldwide public health burden in the majority of countries and are characterized by excessive fat accumulation and, thus, weight gain [1]. Reduction of excess body fat corresponds to the primary goal of a guideline-compliant obesity therapy [2]. Nevertheless, conventional therapeutic approaches that focus on dietary modifications and increased physical activity typically fail to achieve long-term reductions in body fat, even among highly motivated patients [3]. Bariatric surgery offers a promising therapeutic approach, but it is a highly invasive procedure with risks and cannot be the solution for the increasing number of obese people [4,5]. Historically, antiobesity drugs (AOM) have been limited by poor efficacy and significant safety concerns [5]. Many agents were withdrawn after approval due to adverse cardiovascular effects (sibutramine [6]) or psychiatric risks (serotonin agonists [7]). In addition, AOMs predominantly function through either peripheral or central pathways governing energy balance, but rarely both, which fails to adequately address the heterogeneous causes of obesity [5]. These limitations have long hindered sustained therapeutic progress. Incretin-based agents such as glucagon-like peptide-1 receptor agonists (GLP-1 RA) represent a new class of AOMs that were initially used to treat type 2 diabetes mellitus (T2DM) [8]. However, compelling empirical observations offer an effective approach as an AOM to counteract the global rise in obesity prevalence [9,10].

GLP-1 is synthesized by the L-cells in the gastrointestinal tract (GIT) when food, particularly glucose, is ingested [11]. The physiological functions of GLP-1 are manifold, ranging from effects on heart function and the GIT to central nervous system actions, and most importantly, the regulation of insulin secretion in response to blood glucose level [9,12]. Synthetic GLP-1 RAs also affect the central nervous regulation of hunger [13], inhibit glucagon secretion [14], and reduce intestinal motility [15]. This ultimately leads to a significant reduction in food and energy intake, as well as significant weight loss in obese individuals [8]. The first GLP-1 RA approved for weight loss in overweight and obesity, liraglutide, received authorization in the United States (US) market in 2014 [16], and since 2022, 2 GLP-1 RAs (liraglutide, semaglutide) have also been approved for weight loss in European countries [14]. Liraglutide and semaglutide have different pharmacokinetic properties, resulting in different mean weight losses depending on the dose [17]. Higher doses are generally required to address significant weight loss than to improve insulin sensitivity. After a maximum of 68 wk of intervention, the weight loss compared with placebo was 5% with liraglutide treatment [16] and 12% with semaglutide treatment [5,18]. Combining GLP-1 RA with gastric inhibitory polypeptide (GIP) RA, like tirzepatide, leads to a higher mean weight loss compared with placebo, ranging from 12% to 18% after 72 wk of intervention [5,18]. Overall, the treatment with GLP-1 RA leads to a degree of weight loss that has previously only been achievable through bariatric surgery [5]. However, wide variability in the response to GLP-1 RA has been reported in terms of glycemic control and total body weight loss. Up to 30% of patients did not achieve a minimum weight loss of 5% compared with baseline, and identifying predictors of response to GLP-1 RA remains challenging and a clinically relevant issue [19]. In bariatric surgery, preoperative micronutrient deficiencies are the strongest predictor of postoperative deficiencies and are linked to adverse surgical outcomes [20]. It is unclear whether considering dietary quality and micronutrient status prior to GLP-1 RA could improve the response to GLP-1 RA.

In addition, data on the long-term effects of GLP-1 RA on weight reduction remain limited, although sustained effects have been observed for ≤ 4 y [21,22]. Rodriguez et al. [23] recently reported that, within a cohort of 125,475 obese patients, 46.5% of those with T2DM and 64.8% of those without T2DM discontinued GLP-1 RA within 1 y. Discontinuing GLP-1 RA leads to substantial long-term weight regain, ranging from 60% to 163% of prior weight loss, depending on the type of GLP-1 RA [24]. However, the impact of early discontinuation and subsequent weight regain on dietary behavior, including the potential for disordered eating or (micro)nutrient imbalances, remains unclear. In 2024, 6% of US adults reported current use of GLP-1 RA, rising to 22% among those clinically diagnosed with overweight or obesity [17]. However, it is unclear to what extent and with what consequences individuals without prior medical treatment or formal diagnosis related to overweight, obesity, or diabetes utilize GLP-1 RA.

The significant weight loss under GLP-1 RAs and reduced food intake makes it crucial to consume nutrient-dense foods in order to achieve adequate nutrient intake, particularly with regard to micronutrients such as vitamins and minerals [25]. Due to the substantial weight loss over a short period, GLP-1 RA may cause a disproportionate decrease in micronutrient intake, especially when energy intake falls < 1200 kcal/d for women and 1800 kcal/d for men [26,27]. Many individuals who want to lose weight follow a nutritionally inadequate diet, which increases risk of not meeting the recommended intake of several micronutrients, including iron, calcium, magnesium, zinc, and vitamins A, E, K, B1, B12, and C [25,27]. This could further compromise their nutritional status [28]. In addition, obese individuals are already at a higher risk of micronutrient deficiencies, due to unbalanced diets, reduced bioavailability driven by inflammation (iron [29], vitamin B6 [30]), inflammation-driven alterations in binding proteins that lower circulating concentrations of certain micronutrients, and reduced sun exposure (vitamin D) [20,31,32]. Indeed, a high frequency (≥ 50%) of lower than recommended intakes of iron, calcium, folic acid, and magnesium was found despite a high-caloric intake in an obese population [20]. Beyond the lower dietary quality of obese populations, electrolyte disturbances are reported in very low-calorie diets or during rapid weight loss phases [33]. Furthermore, very low-calorie diets could further compromise the micronutrient status of obese individuals [34]. In addition to micronutrient status, the loss of muscle mass may be significantly higher when using GLP-1 RA than with traditional weight loss programs involving diet and exercise [35,36]. Therefore, in addition to nutrient density, a higher protein intake may be necessary to maintain a positive protein balance.

## There is Still a Lack of Data

There is still a lack of data on whether GLP-1 RAs could negatively impact dietary patterns, which in turn affect micronutrient status and body composition. According to medical guidelines, pharmacological treatment for obesity is only initiated after basic therapies, such as nutrition, exercise, and behavioral counseling, have failed to achieve significant weight loss. It is important to note that pharmacological treatment is always started alongside the continued implementation of these basic therapies [2]. However, in clinical practice, the extent to which basic interventions are consistently maintained alongside medication remains uncertain. In addition, there is currently no consensus on the dietary reference intake for low-calorie diets. A recent review by Mozaffarian et al. [17] states that many individuals prescribed GLP-1 RA have not received meaningful nutrition or other lifestyle counseling preceding, accompanying, or after the treatment. This guidance from 4 scientific organizations recommends carrying out a full assessment of dietary history and food frequencies to assess diet quality using food scores [17]. As a relevant comparison, an investigation by Poli et al. [37] showed that, even under long-term interdisciplinary therapy with nutritional counseling, patients who have undergone bariatric surgery do not achieve adequate intake of several micronutrients. Almandoz et al. [25] recently reported specific nutritional recommendations for individuals undergoing GLP-1 RA, addressing fluid, energy, protein, and fiber intake, but not micronutrient intake.

To date, only a few studies have investigated the effect of GLP-1 RA on nutrient intake and status, particularly with regard to micronutrients. Johnson et al. [36] conducted a cross-sectional study in which they used 3-d food diaries to assess the nutrient intake of 69 subjects who had been receiving GLP-1 RA for between 1 and >1 y. Of these subjects, 53.6% were taking semaglutide, 33.3% were taking tirzepatide, 11.6% were taking dulaglutide, and 1.4% were taking liraglutide. Since starting GLP-1 RA, subjects reported eating more protein, fruits, vegetables, and dairy foods. However, the total energy intake (1,748 kcal/d) was higher than expected, with calories from carbohydrates accounting for 41.5% of total energy, calories from fat accounting for 39.9%, and calories from protein accounting for 18.5%. The author reported an insufficient intake of dietary fiber and several key micronutrients, including calcium, iron, magnesium, potassium, choline, and vitamins A, C, D, and E, whereas saturated fat and sodium were consumed in excess. Adequate intake was achieved for B vitamins, copper, phosphorus, selenium, and zinc. In this context, it is important to note that assessment of dietary intake during food diaries is limited as it is self-reported and, particularly obese subjects, are suspected to under-report dietary intake. As food intake declines under GLP-1 RA, fluid consumption correspondingly decreases, leading to modulation of fluid homeostasis and electrolyte equilibrium in human body [33]. Moreover, GLP-1 RA can be associated with several adverse effects, including vomiting, diarrhea, hypodipsia, and constipation, all of which may further compromise (micro)nutrient status [17]. For semaglutide (2.4 mg doses), the prevalence of common side effects is high, with 44% experiencing nausea, 24% vomiting, 30% diarrhea, 24% constipation, and 20% abdominal pain [17]. Although most side effects are reported to decrease after initial dose escalation. Possible side effects aside, the potential impact of GLP-1 RA on the composition, richness, and diversity of the gut microbiota remains insufficiently studied, with a particular paucity of evidence from human studies [38]. Results from animal studies suggest that the effects differ between GLP-1 RA agents. However, human evidence that gut microbes produce vitamins that are bioavailable and contribute significantly to host physiology is limited and inconsistent [38].

## Initial Studies Show the Change in Food Choice

Beyond that, initial studies have examined how GLP-1 RAs affect food choices and behaviors. In a recent review, Christensen et al. [33] reported that GLP-1 RA reduced caloric intake by 16 to 39%. The author highlighted that the observed reduction in dietary intake was based on meals prepared under standardized conditions, which likely does not accurately represent overall macro- and micronutrient consumption. A few recent studies suggest that preferences for certain foods change over time under GLP-1 RA, and that reductions in energy intake are not evenly distributed across daily meals. Almandoz et al. [25] suggest that GLP-1 RA, such as semaglutide and tirzepatide, may influence food preference toward healthy eating patterns. Kadouh et al. [39] observed a significant reduction in total food intake, as well as a decrease in the preference for sweet, salty, fatty, or savory foods, and an increase in perceived satiety after a standardized liquid breakfast after >16 wk of liraglutide treatment in obese individuals. This was associated with notable weight and body fat reduction. Heise et al. [40] in their study on 109 subjects with T2DM, demonstrated a significant reduction in energy intake at an ad libitum buffet lunch following 8 wk of tirzepatide and semaglutide treatment, with corresponding weight loss (tirzepatide −11.2 kg; semaglutide −4.3 kg). However, they did not observe changes in food preferences or micronutrient intake. Similarly, Martin et al. [41] reported a decrease in energy intake at lunch following tirzepatide administration in 55 obese individuals and observed a shift away from less favorable food choices (e.g., sweets, refined carbohydrates, and fast food) toward fruits, vegetables, and high fat products. However, they did not examine macronutrient or micronutrient consumption in detail. Friedrichsen et al. [42] found a 35% reduction in energy intake compared with placebo at an ad libitum lunch after 20 wk of semaglutide (2.4 mg) treatment in obese participants, alongside a reduced craving for salty, sweet, and dairy foods, using a Control of Eating Questionnaire, but again, no detailed micronutrient intake analysis was performed. At this time, the evidence is insufficient to conclude that GLP-1 RA changes eating behaviors. Some isolated observations suggest that GLP-1 RAs lead to reduced consumption of highly processed and sweetened foods and snacks [43,44]. However, no study observed a shift toward smaller meal sizes and an increased focus on consuming more nutrient-dense and fortified foods. This would ensure the recommended daily intake of certain micronutrients, despite a lower overall food intake.

Given these alterations in eating patterns, concerns have been raised that GLP-1 RAs may exacerbate pre-existing eating disorders or contribute to disordered eating behaviors in susceptible individuals [45]. Long et al. [46] observed that the occurrence of nutritional disturbances such as food craving, food aversion, appetite disturbances, and reduced fluid intake differed between the GLP-1 RA used, showing that exenatide, liraglutide, and semaglutide are more likely to be associated with nutritional disturbances. In contrast, Xie et al. [47] evaluated the effectiveness and risks of GLP-1 RA in the US Department of Veterans Affairs healthcare databases containing data on 215,970 GLP-1 RA users. After a median follow-up period of 3.7 y, they found a significantly lower risk of eating disorders, such as bulimia, and a nonsignificant higher risk of nutritional deficiencies, which were not further defined.

## Consequences

Although the use of GLP-1 RAs is becoming increasingly common, their effect on (micro)nutrient status has rarely been discussed to date. The current data are insufficient and do not allow any conclusions as to whether and to what extent the risk of micronutrient deficiencies could increase. Additionally, the impact of reduced fluid intake should be considered, as fluids like mineral water can be relevant source of minerals such as magnesium and calcium. In addition, future research is needed to systematically investigate the long-term nutritional consequences of GLP-1 RA, paying particular attention to micronutrient intake and status. Based on these findings, targeted strategies should be developed to successfully prevent potential micronutrient deficiencies. Furthermore, paying more attention to micronutrient deficiencies prior to treatment may help to explain the wide variability in glycemic control and total body weight loss observed in obese patients. Bariatric surgery patients receive comprehensive nutritional support, including close monitoring and micronutrient supplementation before surgery, during their hospital stay, and postsurgery. In contrast, patients undergoing GLP-1 RA, despite achieving similar weight loss, often do not receive the same level of nutritional management. They may only visit their healthcare provider for follow-up prescriptions. Even more concerning is the widespread availability of weight-loss drugs on various online platforms, which offer the opportunity to use GLP-RA without professional assistance. Current findings do not allow for formal scientific recommendations regarding supplementation for all users of incretin mimetics. However, reduced food intake suggests that it is hardly possible to ensure an adequate supply of all micronutrients under these conditions. For this reason, until proven otherwise, the administration of a physiologically dosed multivitamin-mineral supplement should be considered, and, as far as possible, nutrient status should be checked regularly.

## Author contributions

The authors’ responsibilities were as follows – FK: conceptualization, systematic search, study selection, drafting the manuscript; KK: conceptualization, study selection, critically editing the manuscript; ME: study selection, critically editing the manuscript; AH: conceptualization, study selection, critically editing the manuscript, supervision; and all authors: read and approved the final manuscript.

## Data availability

This perspective does not utilize original data. For additional details about the data from the referenced studies, consult the original articles.

## Funding

The authors reported no funding received for this study.

## Conflict of interest

The authors report no conflicts of interest.
